# BGData - A Suite of R Packages for Genomic Analysis with Big Data

**DOI:** 10.1534/g3.119.400018

**Published:** 2019-03-20

**Authors:** Alexander Grueneberg, Gustavo de los Campos

**Affiliations:** * Department of Epidemiology and Biostatistics, Michigan State University, East Lansing, MI 48824; † Institute for Quantitative Health Science and Engineering, Michigan State University, East Lansing, MI 48824; ‡ Department of Statistics and Probability, Michigan State University, East Lansing, MI 48824

**Keywords:** big data, parallel computing, distributed computing, genetic analyses, biobank

## Abstract

We created a suite of packages to enable analysis of extremely large genomic data sets (potentially millions of individuals and millions of molecular markers) within the R environment. The package offers: a matrix-like interface for .bed files (PLINK’s binary format for genotype data), a novel class of linked arrays that allows linking data stored in multiple files to form a single array accessible from the R computing environment, methods for parallel computing capabilities that can carry out computations on very large data sets without loading the entire data into memory and a basic set of methods for statistical genetic analyses. The package is accessible through CRAN and GitHub. In this note, we describe the classes and methods implemented in each of the packages that make the suite and illustrate the use of the packages using data from the UK Biobank.

Modern genomic data sets are typically big (large-N), high-dimensional (each subject may have information on potentially millions of variables) and multi-layered (*e.g.*, multi-omic data). Storing, handling and analyzing such data sets can be extremely challenging. Most Scientific Computing Environments (SCE, *e.g.*, R, Python, Julia) offer data structures that can handle big data; however, these formats are often not tailored to genetic data. Moreover, data stored in popular genetic data formats (*e.g.*, the PLINK’s .bed format) cannot be readily accessed for computations from a SCE without loading all the data into physical memory. This imposes serious limitations when analyzing very large genomic data sets such as the ones emerging from modern biobanks (*e.g.*, the UK Biobank, http://www.ukbiobank.ac.uk).

The BGData suite of R (R Core Team 2018) packages was developed to offer scientists the possibility of analyzing extremely large (and potentially complex) genomic data sets within the R environment. The suite currently includes four packages (Table 1) which together offer: **(i) a matrix-like class** that enables access to genotype data stored in a PLINK (Purcell *et al.* 2007; Chang *et al.* 2015; Purcell and Chang) **.bed file** without reading the entire file into physical memory, **(ii)** the novel concept of **linked arrays** that allow users to link data contained in different objects into a single array without merging the content of the sub-arrays, **(iii) computational methods** based on the *split-apply-combine approach* that exploit parallelization (multi-core and multi-node)^2^ and enable computations on linked arrays without loading the entire data into memory, and **(iv)** a set of methods for standard **computations with genomic data**, including summary statistics, genome-wide association analyses, computation of genomic relationships, etc.

**Table 1:** Packages, their purpose and repositories

Name	Purpose	GitHub Repository^1^
BEDMatrix	Matrix-like class to extract genotypes from PLINK .bed files	https://github.com/QuantGen/BEDMatrix
LinkedMatrix	Matrix-like class to link matrix-like objects by rows or by columns	https://github.com/QuantGen/LinkedMatrix
symDMatrix	Matrix-like class to link blocks of matrix-like objects into a partitioned symmetric matrix	https://github.com/QuantGen/symDMatrix
BGData	Computational methods for matrix-like objects, a class to represent genotype/phenotype data, and the flagship package of the suite	https://github.com/QuantGen/BGData

All packages are also available at https://CRAN.R-project.org/.

The data structures and methods included in BGData allow users to work on very large arrays (of potentially different data types) with sections (*e.g.*, sets of columns or sets of rows) stored in different physical locations (*e.g.*, multiple .bed files) without physically merging the data. In this article, we introduce the main classes and methods implemented in the BGData suite of packages and illustrate their use by implementing a pipeline for analysis of data from the full release of the UK Biobank.

## Materials and Methods

The suite is currently composed of four packages (Table 1). The flagship package is BGData; the whole suite can be installed by installing BGData from either CRAN
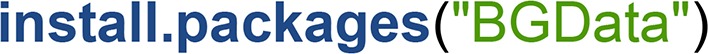


or GitHub, in which case the devtools package (Wickham *et al.* 2018) must be installed and loaded in advance.
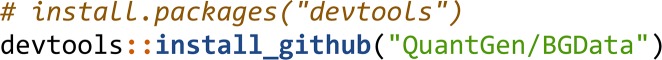


**An example data set**, a subset of the *Arabidopsis thaliana* 250k SNP and phenotype data (Atwell *et al.* 2010), is included with the BGData package. The genotype data were split into chromosomes and converted to PLINK .bed binary files and the accompanying .bim and .fam text files. Only the first 300 SNPs of the first three chromosomes were included to keep the size of the dataset small enough for package distribution. FT10 has been chosen as a phenotype and is provided as a text file called pheno.txt. The data are included in the extdata folder of the BGData package.
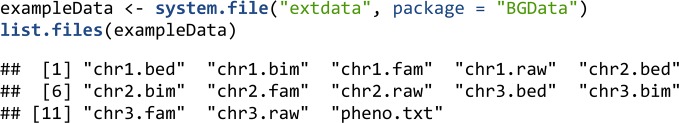


### Matrix-Like types

Phenotypes, genotypes and data from many omics can be stored in two-dimensional arrays. Core R has a limited selection of such types (*e.g.*, matrix, data frame) and their instances are stored in physical memory. Holding data in physical memory allows for very fast computations but is constrained to small data sets. Genomic data sets often exceed those boundaries. However, many operations involved in genetic analyses can be performed on chunks of the data; therefore, not all data needs to be loaded into memory at the same time. There are several packages that implement “matrix-like” classes that extract chunks from binary flat files on disk on demand rather than keeping the entire file in memory, *e.g.*, ff (Adler *et al.* 2018), bigmemory (Kane *et al.* 2013), mmap (Ryan 2018), and filematrix (Shabalin 2018). These classes have an interface like the matrix class of R: there are methods to extract data (using square brackets), get the dimensions of the matrix (using dim()), get the length of the underlying array (using length()), and update row and column names (using dimnames()). We extend the functionality available in R for matrix-like objects by contributing three novel classes: BEDMatrix, LinkedMatrix and symDMatrix.

### BEDMatrix

We developed the **BEDMatrix package** to offer a class and methods for memory mapping of PLINK .bed files. Memory mapping is a technique that maps an entire file or a part of it into the virtual memory of a process. Pages of this mapping can be accessed on demand and the kernel retrieves the requested pages efficiently from disk. The BEDMatrix() function takes the path to the .bed file as an argument and returns a BEDMatrix object.
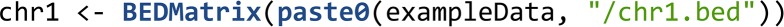


Compared to .ped files, PLINK’s plain-text representation of genotypes, .bed files are compact and therefore suitable for very large datasets. The binary .bed files do not store metadata such as the number of samples and the number of variants; therefore, they are accompanied by a .fam file that describes the samples and a .bim file that describes the variants. Both files must be in the same directory and have the same root name as the .bed file.

Attributes from a BEDMatrix object can be accessed using the same functions used for regular matrix objects, namely, dim(), length(), and dimnames().
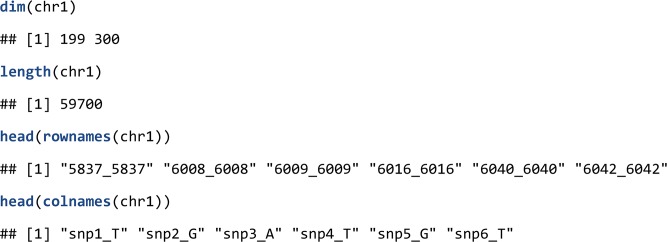


Note, that the creation of the object only establishes the memory mapping, the actual data are not loaded in physical memory. However, users can extract subsets of the data as regular matrix objects that live in physical memory using indexing operations.
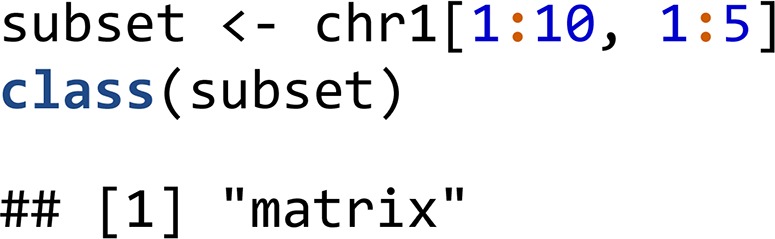


Replacement is not implemented for BEDMatrix; therefore, the content of the .bed file cannot be altered from within R. We believe that this task is better left to PLINK to reduce accidental writes to the file and to consequently increase reproducibility of analyses.

### Linked arrays

Very large genomic data sets are often stored in multiple files. For instance, large genome-wide association studies (GWAS) data sets are often stored by chromosome. On the other hand, data from consortia and many data sets in animal and plant breeding are stored in multiple files, each of which contains a subset of the subjects (*e.g.*, cohorts). To enable analysis of data stored in multiple files, we developed the concept of linked arrays. The **LinkedMatrix package** contains classes to virtually link matrix-like objects by rows or by columns. RowLinkedMatrix is a matrix-like type that links matrix-like objects by rows, and ColumnLinkedMatrix is a matrix-like type that links matrix-like objects by columns. Both classes are subclasses of LinkedMatrix. The following example loads the remaining two chromosomes and links all three chromosomes by columns:
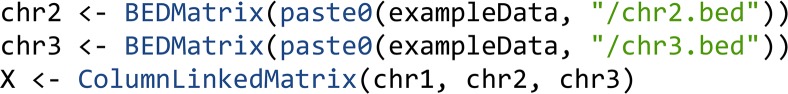


LinkedMatrix objects behave similarly to regular matrices; thus, one could potentially link by columns or rows various linked arrays into a ‘meta-array’. Importantly, LinkedMatrix can link arrays of different types. For instance, one could link by columns a BEDMatrix containing genotypes and a data frame with phenotypes (of course, such operation will be meaningful provided that both arrays have the same number of rows and are sorted accordingly).

LinkedMatrix objects are themselves matrix-like types that can be linked with themselves, a technique illustrated in the symDMatrix package.

#### Creating linked arrays from a list of files:

The RowLinkedMatrix() and ColumnLinkedMatrix() constructors behave similarly to the list constructor and can be combined with the do.call() function. Alternatively, the as.ColumnLinkedMatrix() method for lists can be used to initialize a linked array from a list of matrix-like objects.
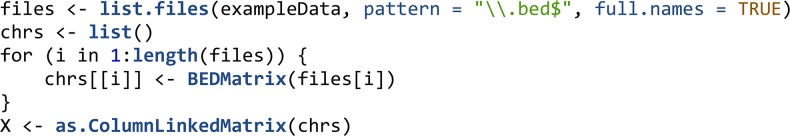


### Efficient computational methods for file-backed arrays

Neither BEDMatrix nor LinkedMatrix have specialized computational methods. This is addressed in the **BGData package**. While the methods implemented in BGData were originally developed with a focus on file-backed arrays, they can be used with any matrix-like object (*e.g.*, including regular matrices, data frames and sparse matrices).

The computational methods implemented in BGData follow the **split-apply-combine approach** (Wickham 2011): chunks of the array (either columns or rows) are loaded into memory and computations on these chunks are performed using standard R functions. Results from each of these chunks are either combined or aggregated. For instance, suppose we want to compute the matrix product XY’ without fully loading X and Y in memory. The operation can be reduced into sub-tasks that can later be aggregated $XY^{’}=\overset{p}{\underset{j=1}{\sum }}X_{j}Y_{j}^{’}$. Here, $X_{j}$ and $Y_{j}$ are columns of $X=\left[X_{1},X_{2},...,X_{p}\right]$ and $Y=\left[Y_{1},Y_{2},...,Y_{p}\right]$, respectively. Similar concepts can be used for implementing ‘apply-like’ operations.

#### Apply-function for file-backed arrays:

The chunkedApply() function is similar to apply() and allows to call a function on each row or column of X, depending on MARGIN (1 for rows, 2 for columns). chunkedApply() loads either row or column chunks in memory, processes them using methods for in-memory arrays and aggregates the results. The amount of data that is loaded in memory is controlled by the chunkSize parameter. Care must be taken to not set chunkSize too high to avoid memory shortage. The following snippet computes the column means of our linked file-backed X matrix in parallel on four cores, loading 250 columns into physical memory on each core.
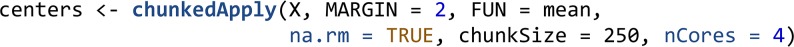


#### Parallel computing:

Most of the functions in BGData support multi-core computing; this is implemented using functionalities offered by the parallel package. The argument nCores (see example immediately above) determines the number of cores the code is run on. In this context, chunkSize determines the amount of data that is loaded in memory per core. For further details on how to specify this argument and others that control how computations are distributed in cores see the online documentation.

#### Computations on subsets of a file-backed array:

Often one needs to carry out computations on a subset of an array only. For instance, one may want to carry out a GWAS on a subset of the subjects (*e.g.*, for sex- or ethnicity-**stratified analyses**). Subsetting a very large file-backed array can be time consuming, memory intensive, and, if the data are written to the file system, lead to data duplications that increase storage needs. Likewise, for distributed computing (*i.e.*, computations distributed over many nodes in a computer cluster) one may want to split the computations on subsets of columns. For instance, a GWAS can be split into tasks, each involving analyses in one chromosome. To this end, most of the functions in the BGData package have two arguments (i, for indexing rows, and j for indexing columns) that allow users to specify rows and columns of the array to be used in computations. By default, these arguments are set so that computations are carried out on the entire array. In a computer cluster, tasks can be distributed over nodes and subsequently combined. The following example illustrates the use of index sets in the chunkedApply() function.
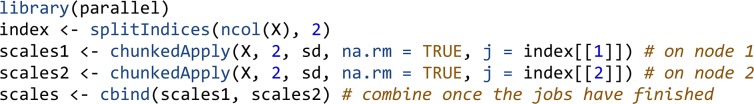


#### The BGData class:

To treat genotypes and phenotypes as one unit, we created the BGData class that contains three slots, modeled after PLINK’s .bed, .fam, and .bim files: geno, pheno, and map. The geno slot contains genotype calls, pheno contains sample information (including phenotypes), and map contains variant information. Typically, geno will be a (potentially linked) file-backed array and the other two would be data frames. When a BGData object is used for computations (see next section) the methods assume that the rows of pheno are in the same order as those of geno. Likewise, the rows of map are assumed to be in the same order as the columns of geno. The following snippets illustrate two ways of creating a BGData object, the first one from either a BEDMatrix object or a LinkedMatrix object containing BEDMatrix objects, the second one from arbitrary matrix-like objects.
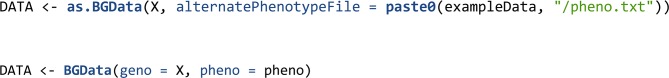


#### Basic functions for common statistical genetic analyses:

BGData also provides some basic functionality for statistical genetic analysis, including computation of summary statistics from genotypes, single marker regression and computation of genomic relationships. All these functions support parallel and distributed computing in the same way as chunkedApply().

The summarize() function computes the minor allele frequencies, frequencies of missing values, and standard deviations of genotypes (*i.e.*, per column of DATA@geno).
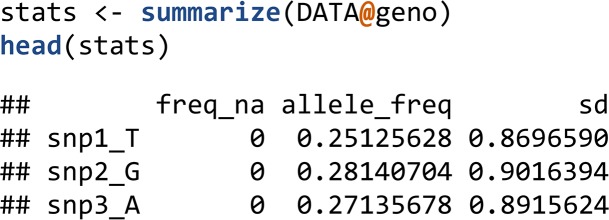


The GWAS() function supports many methods for single marker regression, including least-squares, logistic and probit regression, and mixed models (see the online documentation for a full list), and makes use of a formula interface, which takes the markers from the geno and the phenotypes from the pheno slot of the BGData object passed as the data parameter. The following example uses the rayOLS method to perform a GWAS for the trait FT10 using an intercept plus one SNP at a time model.
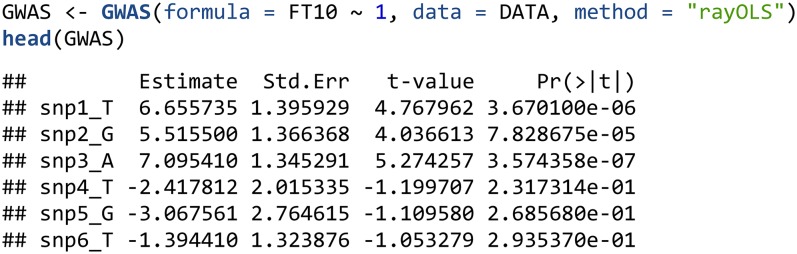


For least squares we offer three methods: lm, lsfit and rayOLS. The first two invoke the corresponding R functions (note that lsfit is often much faster than lm). For models without covariates (specified as trait∼1 in the GWAS function), rayOLS is the faster option; it centers both the phenotype and the variants and regresses the centered phenotype on the centered marker using a model without intercept.

The getG() function computes a positive semi-definite symmetric genomic relation matrix XX’ offering options for centering and scaling the columns of X beforehand.
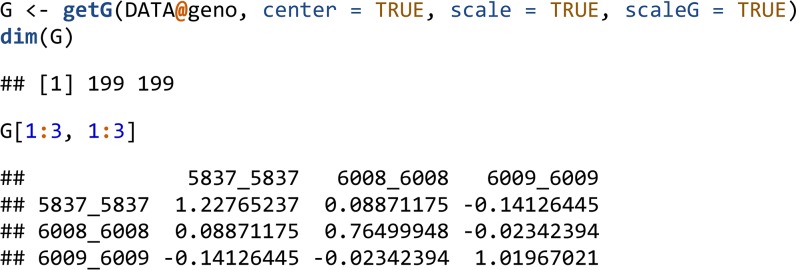


For matrices with a large number of individuals, genomic relationship matrices can be prohibitive to hold in memory. The symDMatrix class of the symDMatrix package was developed to represent a symmetric matrix (*e.g.*, XX’) partitioned into file-backed blocks. The symDMatrix class is a special case of a LinkedMatrix and inherits from the RowLinkedMatrix class. It can store instances of the ColumnLinkedMatrix class containing blocks of file-backed ff objects. The lower-triangular blocks can be efficiently stored as virtual transposes of the upper-triangular blocks. Internally, getG_symDMatrix() uses getG() with an i2 argument to control the individuals of Y. getG_symDMatrix() supports the same centering and scaling options as getG().
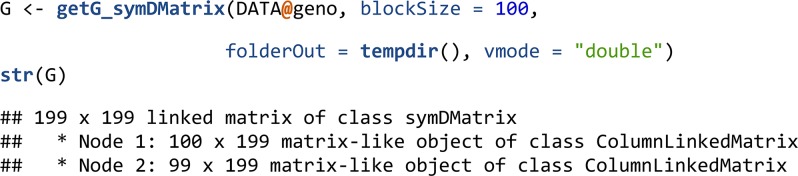


### Developing new functionalities

New functionality for algorithms involving computations one-variant or one-sample at a time can be easily developed with the chunkedApply() function. For instance, suppose one has a function that computes a chi-square test for Hardy-Weinberg Equilibrium for a single locus (say, getChisq()). Then, a function that performs the operation in the entire array can be created using
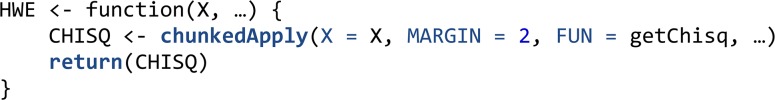


### Data Availability

The packages are available on CRAN and GitHub (Table 1). Supplemental material is available on GitHub at https://github.com/agrueneberg/BGData-Supplemental-Material. The example pipeline uses data from the UK Biobank under project identification number 15326. The data are available to all *bona fide* researchers and can be acquired by applying at https://www.ukbiobank.ac.uk/register-apply/.

## Results

### Example of a Pipeline Combining BGData and BGLR

The examples provided above were developed to illustrate functionality and are based on a relatively small data set that is included in the package. To demonstrate the use of BGData with Big Data we present a modular pipeline (Table 2 and Supplemental File 1) that we used to analyze data from the UK Biobank (n∼500K, K = 1,000). The pipeline combines the use of BGData and BGLR (Pérez and de los Campos 2014) and is inspired by the analyses presented in Kim *et al.* (2017) who applied similar methods for analysis of data from the interim release (n∼150K). Each module in Table 2 completes a task. A brief description of the pipeline follows; further information and example codes can be found in Supplemental File 1.

**Table 2:** Summary of the modular pipeline used to analyze data from the UK Biobank

Task	Data Set		R Package Used
	Train	Test	
1) Form a linked array with genotypes	☒	☒	BGData
2) Determine white British cohort	☒	☒	base
3) Summaries	☒	☒	BGData
4) SNP filtering (allele frequency & call rate)	☒	☒	base
5) Genomic relationships (GR)	☒	☒	BGData
6) Identification of samples with GR < 0.03	☒	☒	BGData
7) Computation of 5 PC	☒	☒	base
8) Phenotypes adjustments	☒	☒	base
9) Building of training and test set	☒	☒	base
10) GWAS (using adjusted phenotypes)	☒		BGData
11) Selection of the top-p variants	☒		base
12) Bayesian Genomic Regression	☒		BGLR
13) Assessment of prediction accuracy		☒	base

The original 26 PLINK .bed files (autosomal chromosomes, sex chromosomes, pseudo-autosomal region of X, and mitochondrial DNA), which contain 805,426 variants for each of the 488,377 samples, were loaded using the BEDMatrix package and linked to a ColumnLinkedMatrix. Phenotypes were loaded and combined with the genotypes in a BGData object.

Inclusion criteria: analyses were based on data of individuals with white British ancestry (determined using the sample QC file, n = 409,637). We produced summary statistics for each SNP using the summarize() function of BGData. Only variants that had minor-allele frequency >1% and calling rate >0.95 (624,523 variants) were used in analyses. Note that we did not create subsets of .bed files. Instead, during the QC process we identified the set of individuals and variants that we will use in analyses and passed the row and column position of these individuals/variants to functions using the i and j arguments discussed above.

A genomic relationship among all the white British samples was computed using the getG() function. This task was split into independent jobs that run on different nodes of MSU’s HPCC. Specifically, we partition the individuals into 21 sets (20 sets with 20K subjects and 1 set with 9,637 individuals); these sets lead to partitions of the genomic relationship matrix into (21 * 22) / 2 = 231 blocks. Each of these blocks were computed using the getG() function, using argument i and i2 to specify the rows and columns of the block. Each block was saved into an ff object and all the blocks were linked into a single array using the symDMatrix class. The getG_symDMatrix() function is a convenience method that performs these steps in an iterative manner. The resulting (linked) genomic relationship matrix was used to determine a set of individuals that had mutual genomic relationships smaller than 0.03 (*i.e.*, distantly related). This was done using the findRelated() function in BGData. For the 233,117 unrelated white British samples, we computed the principal components based on a singular-value decomposition using the svd() function in R and using 5,000 evenly spaced variants.

The standing height phenotype was adjusted by sex, age, batch, assessment center, and the first five principal components using the lm() function in R. Outliers were removed.

Training/testing: the set of unrelated white British samples with complete observations for standing height was randomly split into a test set of 10,000 samples and the remaining 222,648 samples were used as a training set.

**Figure 1:**
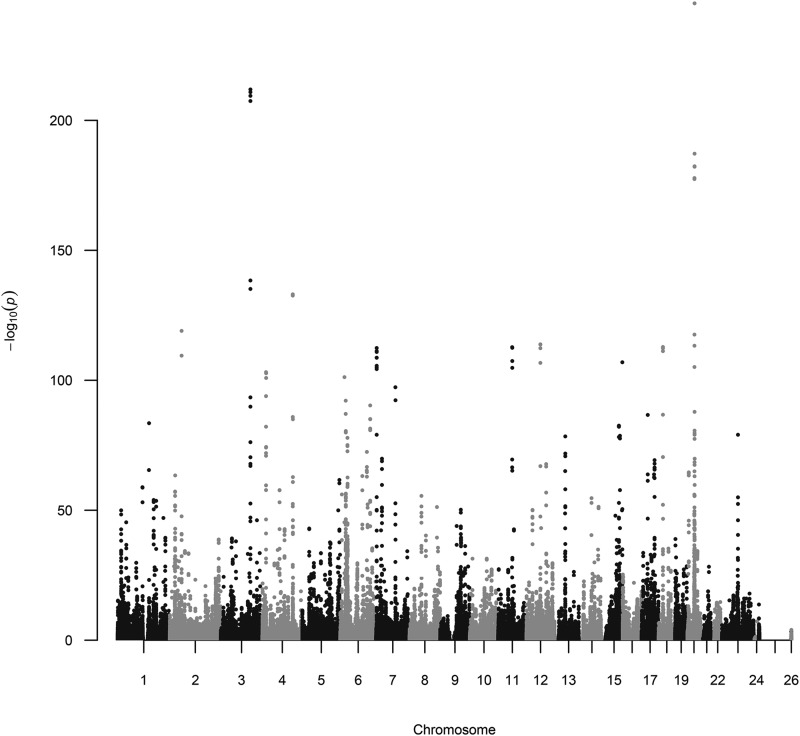
Manhattan plot obtained by regressing sex-age adjusted height on variants using data from the training set (n = 222,648, unrelated White British).

Association analysis was performed on the training set, regressing the adjusted trait on one variant at a time using the GWAS() function and rayOLS as a regression method. Figure 1 shows the resulting Manhattan plot generated with the qqman package (Turner 2014).

**Figure 2:**
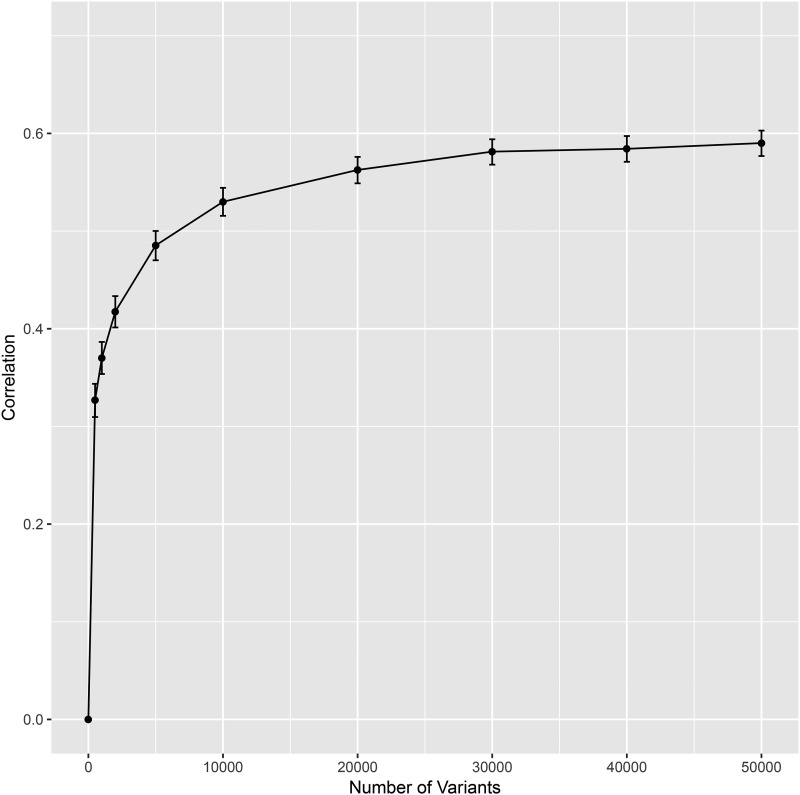
Correlation (+/− SE) between sex-adjusted height and predicted height in the testing set, by the number of SNPs used.

We used the results from the GWAS (Figure 1) to rank variants. Further analyses were conducted using the top-p (*i.e.*, those with smallest p-value, for p = 500, 1K, 2K, 5K, 10K, 20K, 30K, 40K, and 50K, K = 1,000 variants). These variants were grouped into count-based sets and used to fit a Bayesian regression where the adjusted phenotype was regressed on the selected variants using the BayesB method of the BGLR package. The estimated effects were then used to predict height in the testing set, and prediction accuracy was estimated by correlating predicted and observed (adjusted) height (Figure 2). The standard errors in Figure 2 were estimated using bootstrap samples of the vectors of predicted and observed (adjusted) height.

We ran all the tasks on a node with four cores and 350 GB of RAM provided by the MSU High Performance Computing Center (HPCC). A summary of the computational times involved in some of the most demanding tasks of the pipeline is presented in Table 3.

**Table 3:** Computational times involved in some of the most demanding tasks of the pipeline, performed on a node with four cores and 350 GB of RAM provided by the MSU High Performance Computing Center (HPCC).

Task	Dimension	Median Time (SD) in either seconds (s) or hours (hr)
**Summaries**	n = 410K×p = 805K	0.27 hr (23.94 s)
**Block of Genomic Relationship Matrix**	n = 20K×n = 20K	1.00 hr (105.32 s)
**GWAS**	n = 223K×p = 625K	0.57 hr (139.9 s)
**BGLR Regression (single iteration)**	n = 223K×p = 0.5K	0.156 s (0.039 s)
	n = 223K×p = 1K	0.391 s (0.061 s)
	n = 223K×p = 2K	0.612 s (0.042 s)
	n = 223K×p = 5K	1.665 s (0.095 s)
	n = 223K×p = 10K	3.355 s (0.126 s)
	n = 223K×p = 20K	6.858 s (0.174 s)
	n = 223K×p = 30K	10.804 s (0.240 s)
	n = 223K×p = 40K	14.985 s (1.200 s)
	n = 223K×p = 50K	20.901 s (0.536 s)

223K and 625K were n = 222,661 and p = 624,528.

## Final Remarks

We developed BGData to make the analysis of extremely large genetic data sets, potentially stored in multiple files, possible within the R environment. The suite of packages achieves this goal by providing classes and methods to access genetic data sets stored in hard drives without loading the entire dataset into memory. Our approach combines memory mapping (provided by BEDMatrix) with the possibility of (virtually) linking several data sets into a single array using the classes and methods defined in the LinkedMatrix package. We also provide a set of tools for common genetic data analyses, many of which are based on the *split-apply-combine* strategy and incorporate multi-core and distributed computing.

There are a large number of packages for statistical genetic analyses in R that require either genotypes in memory or files in formats other than .bed, *e.g.*, BGLR, synbreed (Wimmer *et al.* 2012), and R/qtl (Broman *et al.* 2003). BEDMatrix opens the possibility to use these packages with .bed files by extracting subsets into memory or files under memory constraints. Recent developments in R Core added support for native memory-mapping and other alternate representations of data (ALTREPs), which may eventually enable packages to make use of file-backed matrices without being optimized for them. The examples provided in this study illustrate how BGData can be used in combination with existing R packages to enable genomic analyses with very large data sets. BGData can be highly synergistic with R packages that implement methods for multi-core and/or multi-node systems.

For routine problems such as summarizing a dataset or performing a genome-wide association study, we have developed a few custom functions in C that carry out the computations faster and using less memory than what is available in R, but the other functions are implemented in R and thus, the performance and memory usage is bound by the performance of the R methods used. In the future, we plan to develop additional custom functions to further improve the performance of tasks that are routinely used such as computations of genomic relationship matrices that are currently bound by the performance of the crossprod() function in R.

For better performance, it may also be worth looking into general-purpose data formats such as HDF5 or column-oriented databases. We have not pursued this because many data repositories such as UK Biobank and dbGaP provide their data in .bed format and the format is simple, efficient and compact enough to not require additional infrastructure to store alternate representations of the same data. In addition, PLINK 2.0 will introduce a new format called .pgen, adding new features such as more efficient indexing, variable-length records, and LD-based compression, which will reduce the file size even more. We are aiming to add support for .pgen once the format has been finalized and widely adopted.

We will further develop and maintain the package both in CRAN and GitHub. In the spirit of open-source software we invite users to develop and contribute new functionality in the form of functions or package that take advantage of the classes and methods defined in the BGData suite of packages.
